# The Goodyear Rubber Cases

**Published:** 1868-11

**Authors:** 


### UNITED STATES CIRCUIT COURT.

### THE GOODYEAR RUBBER CASES.

### DECISION OF JUDGE LEAVITT.

Saturday morning Judge Leavitt pronounced his decision in the case of Henry B. Goodyear vs. A. Berry, J. Taft and about twenty other dentists. This case was an application for a perpetual injunction against the use of a hard rubber for dental purposes, which plaintiff claimed was an infringement on his patent. It was heard in this Court last spring.

Cases involving the same questions have been submitted to the District Judge in the Northern District of Illinois, and to the Judge of the Eastern District of Missouri, and the Court, in the expectation that either the one or the other of these judges would decide the case, has postponed the announcement of its opinion until this time. There were some eight or nine cases submitted at the same time, all involving the same questions and the same facts.

The bill alleges an infringement, by the defendant, of two re-issued patents to Henry B. Goodyear, as administrator of Nelson Goodyear, dated the 18th of May, 1858, for an improvement in making hard rubber or vulcanite. These re-issued patents were extended for seven years from the 6th of May, 1865. The infringement charged consists in the alleged use of hard rubber by the defendants, as plates for the insertion of artificial teeth. The bill prays for an injunction and an account of profits.

The history of the invention covered by complainant's patents is briefly this:

In June, 1844, Charles Goodyear applied for and obtained a patent for an improvement in the process of preparing India rubber, or caoutchouc. In December, 1849, this patent was surrendered. and a re-issue granted on an amended specification. And subsequently another re-issue was obtained. These patents embrace substantially the mode of producing a soft and plastic article known as valcanized rubber, by subjecting the rubber, in combination with sulphur and other ingredients, to a high degree of artificial heat. The article produced by this process was called vulcanized India rubber, and was used for the various purposes contemplated by the inventor.

In May, 1851, Nelson Goodyear obtained a patent for a new and useful improvement in the preparation of India rubber, by which the article known as hard rubber, now extensively applied to many useful purposes, was produced.

The patentee having died, the re-issued patents, numbered 556 and 557—one for the process and the other for the product—were granted to the said Henry B. Goodyear, as administrator of Nelson Goodyear, dated May 18, 1858, and subsequently extended for seven years.

Numerous grounds of defense are set up in the defendants' answer, but those relied on in the argument are as follows:

1. That the re-issued patents to Henry B. Goodyear are void, as not being the same invention as the original.
2. That the re-issues were improperly granted.
3. That the fact that the dentists in this vicinity openly and notoriously purchased and used the soft rubber for making hard rubber plates for artificial teeth, is a bar to a suit in equity; aud that if the complainants have a remedy, it is at law.

4. That no infringement is proved.

Before noticing specially these several grounds of defense, it will be proper to remark that the re issued patents, on which this suit is brought, have heretofore been the subjects of litigation, and have been judicially passed upon. And in so far as principles involving the validity of these patents have been settled by these decisions, they will be regarded as final and authoritative on this Court.

It appears that in the spring of 1861 a suit in equity was brought in the Circuit Court of the United States for the Southern District of New York, by Henry B. Goodyear and Conrad Poppenhizen vs. the New York Gutta Percha and India Rubber Vulcanite Company and others, charging an infringement of both the re-issued patents of H. B. Goodyear, and praying for an injunction and account. The bill was similar in its frame and averments to that filed in the case before this Court. The defendants, in their answer, denied the validity of the re-issues, alleging they were obtained by fraud, and insisting that Nelson B. Goodyear was not the first and original inventor of vulcanite, or the process of making it. The case was elaborately argued before the New York Court, at October Term, 1862; and the Court, Judge Nelson presiding, after mature consideration, sustained the validity of the re-issues, and awarded a perpetual injunction.

In January, 1867, another bill was filed in the same Court, in the name of Henry B. Goodyear and others vs. Thomas G. Waite, alleging an infringement of these re-issud patents, and praying for an injunction. The answer of the defendants, in that case as in this, denied the validity of the re-issues on the grounds of vagueness and insufficiency of the specification; that the re-issues were for an invention different from, and broader than that claimed in the original patent; and, also, that they were void as being for a process and a product in separate patents for the same invention. The infringement charged was also denied by the answer, and it was also alleged, as a ground of defense, that the complainants, by their long acquiescence in the use of hard rubber by dentists for dental purposes, had abandoned their right to the exclusive use of the article for such purposes, and had dedicated it to the dental profession. This case was strenuously contested. A large mass of testimony was taken by the parties, and it was elaborately argued by distinguished counsel, and finally decided by the learned Judge Nelson in August last. This decision was made subsequently to the hearing of the motion for a preliminary injunction in the case now pending. The learned Judge just named, after taking the case under advisement, decided all the points in controversy in favor of the complainants, granting a perpetual injunction, and a decree against the defendants for profits.

The opinion of Mr. Judge Nelson in the case just referred to, is before the Court, and has been carefully noticed. He decides, in substance, the following points:

1. That the description of the invention of Nelson Goodyear, as contained in the two re-issued patents, is sufficiently full, clear and exact to meet the requirements of the statute.

2. That the invention of Nelson Goodyear, consisting of a process for the production of the article known as hard rubber, was original with him, and properly the subject of a valid patent, both for the process and the product; and that Henry B. Goodyear, as the administrator of Nelson Goodyear, had a right to surrender the original patent for an insufficient description in the specification, and to the two re-issued patents granted to him.

That the evidence did not make out a dedication of the right accruing under such issued patents, to the dental profession or the public; and that the use of the hard rubber for dental purposes, by unlicensed persons, might be restrained by the process of injunction, and redress obtained in equity.

The issue of infringement does not appear to have been insisted on by the defendants in the New York case, and there is, therefore, no special finding of the Court upon it. Indeed, it was not denied by counsel in their argument, and from the fact that a decree was entered for the complainants, the irresistible influence is, that the infringement was made out to the satisfaction of the Court.

I have carefully noticed the views and conclusions of Judge Nelson on the points adverted to, and have no hesitancy in adopting them as the views and conclusions of this Court, so far as they apply to the questions and issues now before it. To re-state, at length, the grounds on which the learned Judge placed his decision, would be a useless expenditure of time and labor. They are lucidly set forth in his opinion, and I see no necessity for reproducing them. I shall, therefore, confine myself to the points made in the able argument of the defendants' counsel, not discussed or settled by Judge Nelson.

The first of these points is that the re-issues are void, as being for an invention broader than that claimed in the original patent, and which can not by fair construction be included in it.

This presents a question of great interest to the parties, and, perhaps, not wholly free from difficulty in its solution. I will state the conclusion to which I have been led after careful and anxious consideration.

Nelson Goodyear, in the specification on which his original patent was based, professes to have invented a new and useful improvement in the preparation and manufacture of caoutchouc, or India rubber. He refers to the patent of Charles Goodyear, for the preparation of the plastic compound, and claims to have discovered a new process, by which hard rubber, before unknown, is produced. He describes minutely the ingredients from which, and the process by which the article is produced, disclaiming the invention of the heating or curing process claimed and covered by the patent of Charles Goodyear, and he sums up his invention or claim as follows:

"What I do claim as my invention, and desire to secure by letters patent, is the combining of India rubber and sulphur, either with or without gum shellac, for making a hard and inflexible substance, hitherto unknown, substantially as herein set forth. And I also claim the combining India rubber, sulphur and magnesia or lime, either with or without shellac, for making a hard substance hitherto unknown, substantially as herein set forth."

For the purposes of the question under consideration. it will not be necessary to refer specifically to the description of the process, claimed in the re-issue No. 556, or of the product, as claimed in re-issue No. 557. The claim of the first named patent is as follows:

"What I do claim as the invention of the said Nelson Goodyear, and desire to secure by letters patent, is the combining of the sulphur and India rubber, or other vulcanizable gum, in proportions substantially as specified, according to the vulcanizing process of Charles Goodyear, for the purpose of producing a substance or manufacture possessing the properties or qualities substantially such as described; and this I claim whether the said compound of sulphur and gum be, or be not, mixed with other ingredients as set forth."

The claim of the patent No. 557 is in the following words:

"What is claimed as the invention of the said Nelson Goodyear, deceased, and desired to be secured by letters patent, is the new manufacture or substance herein above described, and possessing the substantial properties herein described, and composed of India rubber or other vulcanizable gum, and sulphur, in the proportions substantially such as described, and when incorporated, subjected to a high degree of heat, as set forth; and this I claim, whether the other ingredients be or be not used, in the preparation of said manufacture, as herein described."

In both these claims the words *other vulcanizable gums* occur, which are not found in the original patent to Nelson Goodyear. In the body of the specifications of each of the re-issues, the words *allied gums* follow after the words *India rubber.* The answer of the defendant sets up these variances between the original patent and the re-issues, and insists that the re-issues are for a different invention from that covered by the original patent, and therefore void. And this view is strenuously urged in the argument of the counsel for defendant.

It may be proper to remark, on this point, that the Commissioner of Patents has passed upon this question, and by the grant of the re-issued patents has given his official sanction to their validity. It is, in effect, his deliberate judgment that the claims of the re-issues are not broader than those of the original, and cover substantially the same invention. It is in accordance with the later decisions of the courts, that the decision of the Commissioner is not conclusive upon the substantial identity of the invention claimed in the original and that claimed in a re-issue. Yet, for reasons not necessary to be stated, his action may well be regarded as affording a presumption in favor of the validity of the re-issue. This presumption, of course, is overcome by evidence of fraud in obtaining the re-issue, or a clear repugnancy between the original and the re-issued patents.

Another remark seems proper in this connection. In the case of Goodyear *et al*, vs. Waite, before referred to as having been heard before Judge Nelson, and decided by him, although this point, as understood by this Court, was distinctly made in the defendant's answer as an objection to the re-issued patents, it was not urged in the argument by his counsel, and was not noticed in the opinion of the Judge. The plain inference from this is that the counsel did not regard it as a sustainable defense, and that such also was the view of the learned Judge who decided it.

The argument of counsel on this point is that the words "other allied gums" used in the specification, and the words "other vulcanized gums," in the claims of the re-issues, are mere interpolations, and impart a claim, not only broader than the original, but which, if they had been inserted in the original, would have invalidated the patent. It is insisted that, in the re-issues, the patentee claims the application of his process to other vulcanizable or allied gums which had not been known, and of which he could, therefore, have no knowledge; and in doing so describes an invention broader than, and differing from, the original.

The doctrine is a familiar one, and well settled, that the invention described and claimed in a re-issue must be the same as originally patented. And, if by a fair construction, the specification and claim are for something substantially different from those of the original, the patent is void. Do these re-issues fall within the scope of this principle?

This is a question of construction, in the consideration of which the entire specifications, including the claims, are to be looked at. And it is well settled by the Courts, that in the effort to ascertain the intention and meaning of the specifications and claims, they are to be viewed in a liberal spirit, that, if possible, the object of the inventor or patentee may be carried out. Mere rigid technicalities are to be set aside, unless there is a clear legal necessity for sustaining them. Now, in reference to these re-issues, there is no pretense that there is any substantial variance from the original, as to the process by which hard rubber is produced, or the character or quality of the article when made. The proportions of rubber and sulphur, and other ingredients, where named, are the same, and the degree of heat is the same. In that which is the very gist of the invention, there is no discrepancy. That consists in the discovery of the fact that the substances named, when compounded as required, and subjected to a certain heat, will produce hard rubber. And now the inquiry is pertinent, whether the suggestion in the claims of the re-issues, that other allied gums, or other vulcanizable gums, may be used to produce the result, can be claimed as changing the character of the invention, or make it broader than the original. Counsel insist that these words enlarge the claim, so as to cover any gum, though then unknown, which may be susceptible of vulcanization. And it may be, if this were the true construction of the claim, the re-issue would be liable to the objection urged. But, clearly, the words referred to are to be taken in a more limited sense. They can be held only to include caoutchouc, and other gums then known to be valcanizable. There would seem to be no impropriety in referring to such; for under the generic term *caoutchouc*, it is well knowm there are different qualities of gums, though all are susceptible of vulcanization. The trees producing it are indigenous in various countries: and owing to peculiarities of soil and climate, the product of the trees differs in quality. The gums, too, are known in commerce by different names, and are of different degrees of purity and excellence, though all produced by trees belonging to the same family. The gum called *gutta percha* has a peculiarity not applicable to any other of these gums. In its natural state it is mixed with woody fibers, and requires a certain preparatory process before it is fitted for vulcanization, or can be applied to any of the uses to which India rubber is suited. Now, by a fair and rational interpretation, are not the words used in the claims of the re-issues, to be limited to these known varieties of the caoutchouc, and not unnecessarily to be extended to all gums thereafter to be discovered? It is a fair presumption that these different qualities of vulcanizable gums were in the mind of the person who framed these specifications, and that there was no intention to anticipate and claim the benefit of future discoveries in that direction. The words used were not, perhaps, necessary to the protection of the rights of the inventor; but in the absence of any proof that they were used for any deceptive purpose, and out of abundant caution, it can hardly be received as such an enlargement of his claim as, in its effect, should invalidate his patent. In a word, I can not view the addition of the words quotod as embracing, within their fair scope, a claim for a different invention from that described in the original patent.

The principle is conceded, that a patent for a mechanical structure or contrivance, producing a new and useful result, is no protection against the use of an invention producing the same result, by appliances and on principles substantially different from the patented invention. The rights of the patentee, or proprietor of the patent, are only invaded by a result like that of his invention, effected by what are substantially the same means. And so in the case of patented chemical combinations; the exclusive right to the invention imports nothing but protection against the use of the same, or substantially the same elements compounded and treated on principles substantially the same as those of the patented article. In brief, a patent right does not cover every possible mode of accomplishing the result proposed by an inventor. And this. as I understand them, is the extent of the decisions of the Supreme Court, cited by the learned counsel for the defendant. The soundness of the doctrine established by that Court is not doubted; but its application to the present case is not so obvious. If the claims of a re-issued patent clearly imply an expansion of the invention beyond the claims of the original patent, there is always ground for a presumption that there was a fraudulent intent to anticipate and protect against subsequent inventions, and thus bar the door against patents for all subsequent discoveries. This is clearly against the policy of our patent right system, and has been wisely condemned by the uniform decisions of the Courts of the United States. This is the import of the decision of the Supreme Court of the United States in *Burr* vs. *Duryea*, 1 *Wallace*, 534, and other cases cited by the defendants's counsel. But I can not see that the claims of these re-issues are of the character which will bring them within scope of these cases.

I have examined attentively the ruling of the Court in the case of *Goodyear* vs. *Providence Rubber Company*, reported in 2 *Fisher*, 499, claimed by counsel to be decisive authority against the validity of these re-issues. That case was before Mr. Justice Clifford, holding the Circuit Court of the United States for the District of Rhode Island, in 1864. The suit was for an infringement of the re-issued patents to Charles Goodyear for his process of making soft or plastic rubber, by vulcanization. The claim of the original patent and the first re-issue was for the process of treating and curing caoutchouc or India rubber; but in the re-issue of 1860, the words "or other vulcanizable gums" were added. And this addition was claimed to be an enlargement of the invention rendering the re-issues void. The question was, therefore, substantially the same as in the pending case. The learned Judge before named construed the claim of the re-issued patent to Charles Goodyear, as including "all other vulcanizable gums," whether then known, or thereafter to be discovered, capable of vulcanization. Viewing the claims in this light, he held that the re-issue was for an invention different from that covered by the previous patents, and therefore void. If this construction of the re-issue was right, probably the conclusion of the learned Judge was correct. But for reasons already stated, I am unable to give the added words in the claims of the re-issued patents, the extended meaning of which he held them to be susceptible, and can not therefore concur with him in his conclusion. It is certainly with some distrust of my own judgment that I differ from that learned Judge; but my convictions are so strong on the question that I do not feel at liberty to yield them, even to his superior learning. If wrong in this, I shall be gratified to have my error corrected by an appeal to a higher Court.

On the question of infringement, there seems to be nothing in the case calling for a minute and extended investigation. In the cases referred to in a previous part of this opinion, establishing the validity of these re-issued patents, the infringement alleged does not seem to have been controverted, and the decrees entered clearly imply that the fact of infringement was made out in Waite's case, the infringement charged was the manufacture and use of hard rubber by dentists as the foundation for artificial teeth. The allegations in the bill were substantially, if not literally, identical with those in this bill, and the answer was the same. And the Court in that case decreed a perpetual injunction.

But the counsel for the defendant in this case takes issue on the question of infringement, insisting that the fact is not technically made out by the evidence. He claims that the defendant denies in his answer under oath, that he has infringed; and that the complainant has proved the fact only by one witness; and, therefore, the Court can not find the fact of infringement. Now, the first remark on this point is, that the defendant's answer does not contain an explicit denial of the infringement. It is evasive in its character. He admits, substantially, the use of hard rubber, made by the process of baking the soft or plastic rubber, for dental purposes, but denies the process is that described and claimed in the re-issued patents. But he adduces no proof that the hard rubber has been, or can be, made by any means or process variant from that covered by the re-issues. And in the absence of such proof, there is at least a fair presumption that the hard rubber used was the article as claimed and described in the re-issues.

And it may also be remarked, that a denial of the infringements is wholly inconsistent with the theory of the defense set up in the answer, and mainly relied on by counsel. That defense is, not that the defendant has not used the hard rubber for dental purposes, but that he had a right to use it, by the implied dedication of such right to the dental profession. This ground of defense is, by a clear implication, an admission of the infringement.

But there is the positive testimony of one witness that the defendant—Berry—and all the other defendants against whom suits are pending in this Court, distinctly admitted the preparation and use of the hard rubber, for dental purposes. As the denial of the infringement in the answer of the defendant is not positive, and the testimony of the witness refered to is strongly corroborated by other facts and considerations bearing on the question of infringement, the fact is made out to the satisfaction of the Court. Upon that issue there is hardly room for a doubt. There is every reason to conclude that hard rubber for dental purposes has been in general use by the profession. It is in evidence that fully three-fifths of the dentists in the United States are licensed under the Goodyear patent; and this fact evinces not only the appreciation of the profession of the article, but its very general use. Those refusing to take licenses evidently intend to place their defense, not on the ground that they did not use the article, but on the ground of the invalidity of the patent, and the dedication of its use to the dental profession.

But it is insisted in the argument of the defendant's counsel, though not set up in the answer as a defense, that the defendant now uses in his profession a hard rubber or compound, made under a patent to Edward L. Simpson, granted October 16, 1866. It is claimed that the process and the product under this patent are essentially different from the claims of the Nelson Goodyear patent, and therefore not an infringement of that patent. This defense, it is obvious, applies only to the issue of infringement. But it is not perceived, if sustained by the testimony, that it is an answer to the claim of the complainants, as made in their bill. If the defendant has infringed by the use of the Goodyear hard rubber, he is liable to account for such infringement, though he may have discontinued the use of the article charged as an infringement of the Goodyear re-issues. If an infringement of the complainants rights under these re-issues is made out, the cessation to use the infringing article is no bar to an injunction, and a decree for an account. I understand the law to be well settled, that, under the circumstances stated, the party whose rights have been invaded may claim protection against future infringements, and is not obliged to rest on the fact that the party has ceased his acts of infringement.

But as the Simpson patent is before the Court, and evidence has been offered, without objection, upon the question of the identity of the process and product under it with that of the Goodyear patent, and the point has been discussed by counsel, it may be expected the Court will pass upon that issue. In noticing this point I shall try to be very brief.

And the first obvious remark in reference to the Simpson patent is, that it does not claim a new process for the vulcanization of India rubber, or that the product is essentially different from that made under the Goodyear patent. In this specification, he says: "The rubber now used for dental purposes has incorporated in its large proportions of free sulphur, for the purpose of vulcanizing the rubber after it is formed." And again: "The color and taste occasioned by the presence of this sulphur is extremely obnoxious to many persons, and occasions the principal, if not the only objection, to the use of rubber for dental purposes. To overcome this objection and produce vulcanized rubber for dental purposes, without the actual or apparent presence of sulphur, is the object of my invention, and consists in preparing the rubber for vulcanizing, by the introduction of a peculiar vulcanizing compound." It is here clearly stated that the object of the patentee was to rid the compound used for dental purposes of the unpleasant taste or odor of the sulphur. He then describes the mode by which he proposes to effect this object, as follows:

"I first boil linseed or other vegetable oil to the consistency of honey, (this I do to facilitate the preparation) thoroughly mix two ounces of benzoin gum with one pound of pulverized sulphur; then to each quart of the boiled oil add one pound of the prepared sulphur, carefully subjecting this mixture to a moderate heat, sufficient only to cause the two substances to react upon each other, until they pass from a semi-fluid to a semi-hard state, having a honeycomb or spongy appearance." He adds, that benzoin gum, "by its vaporizing qualities, more perfectly expels the fumes of the sulphur, as well as the odor from the oil, and renders the compound nearly if not perfectly odorless and when combined with India rubber or similar gum, and subjected to a regulated heat, will cause the same to undergo the change known as vulcanization. In producing rubber for dental purposes, he requires to one pound of India rubber from ten to fourteen ounces of the vulcanizing compound. These are to be thoroughly mixed by being ground between warm rollers, and coloring matter put in if desired. This mixture is plastic, and being rolled into thin sheets, is prepared for use by the dentists. The form of the gums and roof of the mouth being taken in this plastic material in the ordinary mode, it is vulcanized by subjecting it to a heat of 320° of Fahrenheit, for about four hours, or if the heat is above 320°, for a less time. The patentee claims "that the plate thus prepared will be as tasteless and odorless as a metal plate," etc.

The claim of the patent is: "Combining the within described vulcanizing compound with India rubber, in the proportion herein named, and substantially in the manner and for the purposes herein specified." The claim of the Nelson Goodyear patent, and the re-issues under it, have been stated and need not here be reproduced. Now, Simpson, in his specification and claim, does not pretend that by his process he does not produce the article known as hard rubber, or that it is made without the use of sulphur, as required by the Goodyear patent. The claim is, in substance, an improvement upon the known process for its production by the introduction of gum benzoin, to deprive the hard rubber of its sulphurous taste and odor, and thus render it more acceptable for dental purposes. The process of vulcanization is substantially the same as that described in the Goodyear patent, and the product the same, with the exception that it is tasteless and odorless.

As to the identity of the process under the Goodyear and Simpson patent, the complainant has offered the testimony of four learned chemical experts, who have severely tested the elements of the products of both patents, by a rigid analysis. These experiments have been conducted with a view to ascertain the precise ingredients and their proportions, in the compound described in the Simpson patent. Without stopping to state the details of these analysis, as set forth in the testimony of these experts, it is sufficient to say that they harmonize more nearly than can be expected, in the results attained. They find that the compound described by Simpson, when vulcanized, contains about four ounces of sulphur to sixteen ounces of rubber. Thus it is made clear that the article produced under the Simpson claim is vulcanized by essentially the same process, and has very near the same proportions of sulphur and rubber as claimed in the Goodyear patent. These are the vulcanizing agents named in that patent, when subject to the action of heat. The same ingredients and the same process are claimed by the Simpson patent, and the product of the two is essentially the same.

I can have no hesitation, therefore, in holding that the use, for dental purposes, of hard rubber plates, made under the Simpson patent, is an infringement of the Nelson Goodyear patent; and in no aspect of the case is the defendant relieved from liability as an infringer by asserting the use of the product under the Simpson patent. While it is probably true, that Simpson has made a valuable discovery in introducing into his compound an ingredient which, by its vaporizing properties, prevents the unpleasant taste and odor of the vulcanized rubber, when used as plates for artificial teeth, and for this invention may have been well entitled to a patent, he or his licenses are not protected in the use of the process and the product as claimed by and patented to Nelson Goodyear.

I may remark, in closing, that I am fully sustained in this conclusion, by the opinion of Judge Blatchford, District Judge of the United States for the Southern District of New York, in the case of *Goodyear* vs. *Evans*, recently before him, on an application for an injunction to restrain the defendant from the use of hard rubber made under Simpson's patent, as an infringement of the Goodyear patent. In a printed opinion of the learned Judge, now before me, the question is ably discussed, and the conclusion attained that it was a proper case for an injunction, which was accordingly awarded. After noticing the claims of the two patents, and reviewing the testimony as to the infringement, the learned Judge says: "Nothing more is needed to establish clearly that the use of the Simpson vulcanized product is an infringement of Re-issue No. 557, and that the manufacture of it by the Simpson process is an infringement of Re-issue No. 556." Concurring, as I do, in this conclusion, a decree for the complainants will be entered.

John H. B. Latrobe, of Baltimore, T. D. Lincoln, of Cincinnati, and A. Pollock, of Washington, appeared for the complainant; S. S. Fisher, of Cincinnati, for defendants.

